# Supplementary material for: Metastatic breast cancer patient perceptions of somatic tumor genomic testing
Source: BMC Cancer. 2020 May 6;20:389. doi: 10.1186/s12885-020-06905-2 (PMC7201768; doi:10.1186/s12885-020-06905-2)

**Metastatic breast cancer patient perception of somatic tumor genomic testing: Psychosocial factors and genetic comprehension**

Elizabeth J. Adams^2^*, Sarah Asad^2^*, Katharine A. Collier^1,2^, Mahmoud Abdel-Rasoul^4^, Susan Gillespie^2,3^, James L. Chen^1,2^, Mathew A. Cherian^1,2,3^, Anne M. Noonan^1,2,3^, Sagar Sardesai^1,2,3^, Jeffrey VanDeusen^1,2,3^, Robert Wesolowski^1,2,3^, Nicole Williams^1,2,3^, Charles L. Shapiro^8^, Erin R. Macrae^9^, Amanda E. Toland^10^, Leigha Senter^10^, Bhuvaneswari Ramaswamy^1,2,3^, Clara N. Lee^2,6,7+^, Maryam B. Lustberg^1,2,3 +^, Daniel G. Stover^1,2,3+^

**Institutions:** ^1^Ohio State University College of Medicine, Division of Medical Oncology, Columbus, OH, USA

^2^The Ohio State University Comprehensive Cancer Center, Arthur G. James Cancer Hospital and Richard J. Solove Research Institute, Columbus, OH, USA

^3^Stefanie Spielman Comprehensive Breast Center, 1145 Olentangy River Rd, Columbus, OH, USA

^4^Center for Biostatistics, Department of Biomedical Informatics, The Ohio State University, Columbus, OH, USA

^5^Foundation Medicine Inc., 150 Second Street, Cambridge, MA, USA

^6^Department of Plastic Surgery, College of Medicine, The Ohio State University, Columbus. OH, USA

^7^Division of Health Services Management and Policy, College of Public Health, The Ohio State University, Columbus, OH, USA

^8^Mount Sinai, New York, NY, USA

^9^Columbus Oncology, Columbus, OH, USA

^101^Ohio State University College of Medicine, Division of Human Cancer Genetics, Columbus, OH, USA

*Indicates authors contributed equally. +Indicates authors equally directed the work.

**Corresponding Author**:

Daniel G. Stover, MD

Biomedical Research Tower, Room 512

Ohio State University Comprehensive Cancer Center

Stefanie Spielman Comprehensive Breast Center

Columbus, OH 43210

Tel: 614-685-6700

Fax: 614-293-4372

Email: [daniel.stover@osumc.edu](mailto:nlin@partners.org)

**Supplementary Data**

**Supplementary Table 1. Mixed Effects Model**

| **Linear Mixed Effects Model*** | **Estimate (95% CI)** | **Treatment change*time Interaction p-value** |
| --- | --- | --- |
| **CES-D** |  | 0.26 |
| No treatment change |  |  |
| Pre-survey | 11.1 (8.8,13.4) |  |
| Post-survey | 10.8 (8.6,13.1) |  |
| Treatment change |  |  |
| Pre-survey | 17.0 (11.7,22.3) |  |
| Post-survey | 13.5 (7.9,19.2) |  |
| **BAI** |  | 0.29 |
| No treatment change |  |  |
| Pre-survey | 10.1 (8.0,12.2) |  |
| Post-survey | 9.5 (7.3,11.6) |  |
| Treatment change |  |  |
| Pre-survey | 10.8 (5.8,15.8) |  |
| Post-survey | 11.9 (6.7,17.0) |  |
| **TPS** |  | 0.72 |
| No treatment change |  |  |
| Pre-survey | 48.9 (47.3,50.5) |  |
| Post-survey | 49.5 (47.9,51.1) |  |
| Treatment change |  |  |
| Pre-survey | 47.7 (43.8,51.5) |  |
| Post-survey | 47.8 (44.0,51.7) |  |
| **CASE** |  | 0.89 |
| No treatment change |  |  |
| Pre-survey | 43.1 (41.6,44.5) |  |
| Post-survey | 41.9 (40.4,43.3) |  |
| Treatment change |  |  |
| Pre-survey | 44.2 (40.7,47.6) |  |
| Post-survey | 43.2 (39.7,46.6) |  |

*****From linear mixed effects models including main effects for treatment change, time (pre-survey vs. post-survey),

and the interaction term for change and time. Models include random intercepts to account for repeated measures.

**Supplementary Table 2. Patient Motivations and Perceived Risks/Benefits**

| ***Patient Motivations and Perceived Risks/Benefits (Pre-test results, N=58*)*** | | | |
| --- | --- | --- | --- |
| **Finding out that my cancer has a high chance of progressing would be more than I could handle emotionally.** | | | |
| Disagree N(%) | Neutral N(%) | Agree N(%) | |
| 26 (47.3%) | 11 (20.0%) | 18 (32.7%) | |
| **This information about one's cancer is better left unknown.** | | | |
| Disagree N(%) | Neutral N(%) | Agree N(%) | |
| 48 (85.7%) | 6 (10.7%) | 2 (3.6%) | |
| **I am concerned about the test because it is new and hasn't been used widely.** | | | |
| Disagree N(%) | Neutral N(%) | Agree N(%) | |
| 33 (58.9%) | 16 (28.6%) | 7 (12.5%) | |
| **I am concerned that the test being so new prevents me from asking other patients about their experiences with it.** | | | |
| Disagree N(%) | Neutral N(%) | Agree N(%) | |
| 32 (58.2%) | 16 (29.1%) | 7 (12.7%) | |
| **The results will help me change my behaviors and reduce my disease risk.** | | | |
| Disagree N(%) | Neutral N(%) | Agree N(%) | |
| 5 (9.1%) | 19 (34.6%) | 31 (56.4%) | |
| **The results will help me seek medical attention and reduce my disease risk.** | | | |
| Disagree N(%) | Neutral N(%) | Agree N(%) | |
| 2 (3.6%) | 18 (32.7%) | 35 (63.6%) | |
| **I could lose my job if the results get out.** | | | |
| Disagree N(%) | Neutral N(%) | Agree N(%) | |
| 41 (77.4%) | 10 (18.9%) | 2 (3.8%) | |
| **I may learn that I have an increased risk for a disease that I did not want to know about.** | | | |
| Disagree N(%) | Neutral N(%) | Agree N(%) | |
| 30 (53.6%) | 12 (21.4%) | 14 (25.0%) | |
| **I may learn that I have an increased risk for a disease that I can do nothing about.** | | | |
| Disagree N(%) | Neutral N(%) | Agree N(%) | |
| 20 (35.7%) | 14 (25.0%) | 22 (39.3%) | |
| *N per question ranges from 53-56 due to skipped questions | |  |  |

**Supplementary Figure 1. Association of Validated Depression, Anxiety, Physician Trust, and Self-Efficacy Measures with Patient Demographic Features.** Four validated psychosocial measures were assessed in patients at study entry: Center for Epidemiologic Studies Depression Scale (**A;** CES-D)[23], Beck Anxiety Inventory (**B;** BAI) [24], Trust in Physicians/Providers Scale (**C;** TPS)[25], and Communication and Attitudinal Self-Efficacy scale for cancer (**D;** CASE-cancer)[26]. There were no significant differences in any of the validated metrics by income (all ANOVA p>0.05), education (all ANOVA p>0.05), insurance (all ANOVA p>0.05), or breast cancer type (all ANOVA p>0.05).


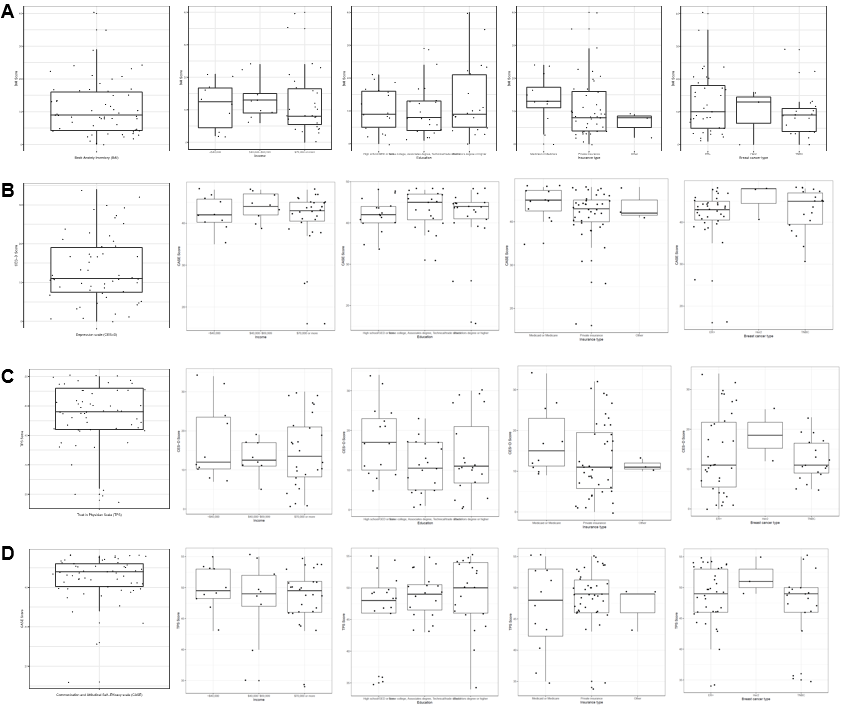

Supplement: Supplementary file 1 — Additional file 1: Table S1. Mixed Effects Model Table S2. Patient Motivations and Perceived Risks/Benefits Figure S1. Association of Validated Depression, Anxiety, Physician Trust, and Self-Efficacy Measures with Patient Demographic Features. [file 12885_2020_6905_MOESM1_ESM.docx]
